# Study on Mechanical Behavior of Hollow-Core Slab Bridge with Pinned Reinforcement

**DOI:** 10.3390/ma16144949

**Published:** 2023-07-11

**Authors:** Jihao Chen, Xin Li, Qian Zhu

**Affiliations:** 1 School of Civil Engineering and Communication, North China University of Water Resources and Electric Power, Zhengzhou 450045, China; cjh@ncwu.edu.cn (J.C.);; 2 School of Civil Engineering and Architecture, Zhengzhou University of Aeronautics, Zhengzhou 450015, China

**Keywords:** hollow-core slab bridges, deep joints, finite element analysis, force transfer mechanism, damage evolution law

## Abstract

Joints connect prefabricated hollow-core slabs, the key elements of force transmission of hollow-core slab bridges. The joints are easily damaged, which affects the integrity and safety of the hollow-core slab bridge. This paper uses MIDAS FEA NX finite element analysis software to simulate the deep hinge joint segment model, comparing and analyzing the finite element simulation analysis results with the test results, and proposes the critical parameters of the hinge joint interface and concrete damage plasticity. Further, an assembled hollow slab bridge model is established to compare and analyze the force transfer performance of conventional and pinned reinforcement bridges and reveal the deep joint damage evolution process. The results showed that the hollow slab bridge damage appeared first at the hinge joint interface at the load location. Cracks in the joints can develop along the longitudinal and height directions, with the longitudinal crack length reaching 40% of the span. The vertical crack height can get the lower edge of the paving layer, increasing the distance from the load position, and the cracking height decreases symmetrically. Under an ultimate load, the hinge concrete of conventional reinforcement and pinned reinforcement hollow-core slab bridges showed significant damage in 30–70% and 40–60% of the span, respectively. Compared with the conventional reinforcement bridges, the cracking load and ultimate load of the pinned reinforcement bridges increase by 28.57% and 58.14%, respectively, and the relative deflection under 420 kN load reduces by 97.95%. The hollow slab bridges have improved the force performance and thus enhanced the integrity of the hollow-core slab bridges.

## 1. Introduction

Creating a solid transportation sector has achieved notable advancements, and the building of transportation infrastructure is in an advanced stage of growth as the nation quickens the pace of creating a powerful socialist modern country. As the primary setting for transportation, bridges have significant importance. Small and medium-sized bridges are the most common type of bridge construction, with many different styles. Assembled hollow-core slab bridges are one type that is frequently used in actual projects because of their advantages [1,2,3], which include low construction costs, an easy construction process, and high manufacturing process standardization [4,5]. The completed hollow-core slab bridge comprises many hollow-core slab girders connected using the post-cast joint. The joints ensure the integrity of the assembled hollow-core slab bridge, making the individual hollow-core slab girders synergistically stressed. Therefore the joints are the key point of the bridge. In the past ten years, bridge damage accidents have occurred frequently, such as the Polcevera viaduct (also known as the Morandi bridge), a highway viaduct in Fossano, a highway flyover in Camerano, a major roadway bridge over the Fiumara Allaro River, and another flyover bridge between Annone and Cesana Brianza [6]. In fact, increasing traffic volumes and aging infrastructure can lead to bridge damage, so it makes sense to monitor existing bridges and improve new ones.

However, in the actual operation process, due to the unreasonable design and construction stage in the bridge operation process, or the increasing overload use and insufficient maintenance, the problems of bridge damage and disease continue to appear. If the joints are destroyed, the transverse connection between the slabs will be weakened [7], and the collective mechanical performance of the hollow slab bridge will be affected [8,9,10]. At this time, the load is concentrated on the single slab, making its force and deformation exceed the average use state, called the single slab phenomenon [11]. It may lead to cracks in the single hollow-core slab and fracture in severe cases [12].

The joint problem attracted the attention of several scholars who had studied the failure process and force transmission mechanism of joints. Brunesi et al. [13] confirmed the key role of the section shape in the shear stress distribution using a combined experiment and FE method-based approach and found that the peak of the shear stress was located at the point below the centroid where the web width changed abruptly. Zhao [14] established a hollow-core slab bridge finite element model to study the stress state of the joints. The analysis showed that tensile stress and shear stress was the leading cause of joint damage when the load acted on the joint.

To enhance the mechanical behavior of hollow-core slab bridges, scholars have studied the effects of different factors on bridges. He et al. [15] investigated the effect of joint concrete strength on bridge bearing capacity, and the results showed that joint concrete strength has a negligible effect on the bearing capacity. Barbieri et al. [16] varied the thickness of the pavement layer and found that increasing the thickness (100, 150, 200, and 250 mm) could reduce the relative displacement of hollow slab beams. Liu et al. [17] analyzed the hollow-core slab bridge by varying the thickness of the pavement layer and found that increasing the thickness could improve the structural load transverse distribution characteristics. Di et al. [18] studied the effect of a post-cast 150 mm thick concrete layer on the bearing capacity and found that the cracking and ultimate load would increase. Liu et al. [19] studied the hollow-core slab beam under flexural load, and the results showed that the bending capacity of the hollow-core slab beam increased by 16.7% after adding the pavement layer. In addition, setting reinforcement in the joints could reduce structural cracking and make the specimens ductile damage [20,21], and increasing the reinforcement rate in the joints enhanced the bearing capacity of the joints [22]. Hanna et al. [23] tested the bearing capacity of specimens with reinforcement in the joints and found that their bearing capacity increased. Ye et al. [24] tested the shear capacity of specimens with different numbers of reinforcements in the joint and found that increasing the number of reinforcements increased the capacity of the specimens by 80.43% and 176.52%. Increasing the joint width also improved the bearing capacity of the joints. Tang et al. [25,26] tested the bearing capacity of specimens with different joint heights, and the results showed that the stress in the joint concrete was reduced when the joint depth was increased from 10 to 26 cm.

With the improvement of technology, finite element software was widely used by scholars in their research. Due to the diverse functions of finite element software, scholars used different methods to establish finite element models of hollow slab bridges. Yuan et al. [27] built XFEM of RC hollow core slabs on ABAQUS, and the rationality of RC hollow core slabs with cracks was further verified. Xiang et al. [28] established a calculation model with a spring-hinged link system and found that the maximum errors of joint shear force, hinge joint transverse force, and hollow slab relative displacement were 0.475 kN/m, 3.832 kN/m and 0.00067 mm, respectively, which verified the accuracy and practicality of the method. Moreno-Padilla et al. [29] proposed a new model that showed a good correlation between the numerical predictions and the behavior observed in reality. Dan et al. [30] established a multibeam model connected by distributed springs to analyze the modal characteristics of a fabricated girder bridge. The analysis of different damage situations proved the applicability of the proposed index. Gui et al. [31] established the finite element models of the beam-shell combination and the articulated beam of the hollow-core slab bridge and found that the mid-span displacement and strain variation of the beam-shell combination model were closer to the measured values, which better reflected the force condition of the hollow slab bridge.

In summary, building finite element modeling of full-size hollow-core slab bridges by finite element software has been widely used by many scholars. Changing the pavement layer thickness or the joints’ form can enhance the bearing capacity of the joints and prevent premature damage. However, few scholars have studied the stress state of the old and new concrete interface of the hollow-core slab joints and the interface damage process during the whole loading process. At the same time, the cracking of the joint surface of the hinge joint is difficult to be observed directly. Moreover, the joint with pinned reinforcement has good mechanical behavior in static load tests [32], but few studies have tested the mechanical behavior of hollow-core slab bridges with pinned reinforcement. In this paper, Midas FEA NX was used to establish the finite element model of assembled hollow-core slab bridge, and proposed the key parameters of concrete damage plasticity and interface unit to investigate the damage of joints in the hollow-core slab bridge, and reveal the joint force transfer performance and interface damage evolution law.

## 2. Experimental Program of Specimens

### 2.1. Specimens Design

**Figure 1:**
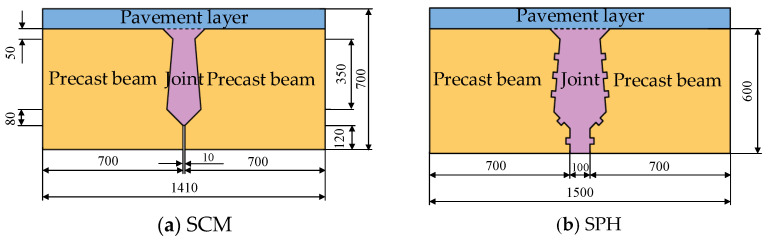
Specimen dimensions and details [32].

The detailed dimensions of the specimens with conventional reinforcement (SCM) and pinned reinforcement (SPH) [32] were as shown in Figure 1. The specimens consist of three parts: post-cast joint, pavement layer, and precast beam. For SPH, wooden blocks were reserved at the interface location to form a recess of size 30 × 20 mm before placing the concrete in the beam section. The hinge joint concrete interface was sprayed with a high-pressure water gun with an average spray depth controlled at 6 mm.

**Figure 2:**
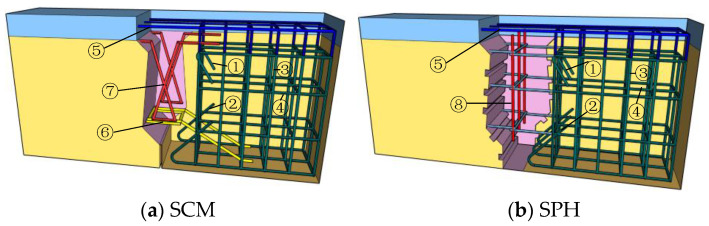
Reinforcement details [32].

The concrete with a design strength of 40 MPa was used for the beam concrete, and the concrete with a design strength of 60 MPa was used for the joint concrete. Hot-rolled ribbed bars with a yield strength of 400 MPa were used for specimens. SPH was also enhanced with hinge joint reinforcement using three layers of U-shaped reinforcement in the form of pin joint reinforcement. The reinforcement details of specimens are shown in Figure 2 [32]. The numbers ①, ②, ③, and ④ represented beam segment reinforcements, and their diameter was 8 mm. The number ⑤ represented pavement layer reinforcements, and its diameter was 10 mm. Moreover, the numbers ⑥, ⑦, and ⑧ represented U-bar, conventional reinforcements, and pinned reinforcements; their diameter was 12 mm.

### 2.2. Test Procedure

In this paper, flexural-shear loading was adopted. The oil pressure gauge was used to control the load value and loading speed during the test. The load was first loaded in increments of 5% of the predicted limit load value until it broke. Then it was continuously loaded in increments of 10% of the expected limit load value. The changes in cracks in the loading process were recorded. The test was terminated when the crack reached the lower edge of the pavement. The load at this time was called the crack penetration load.

### 2.3. Experimental Results

**Figure 3:**
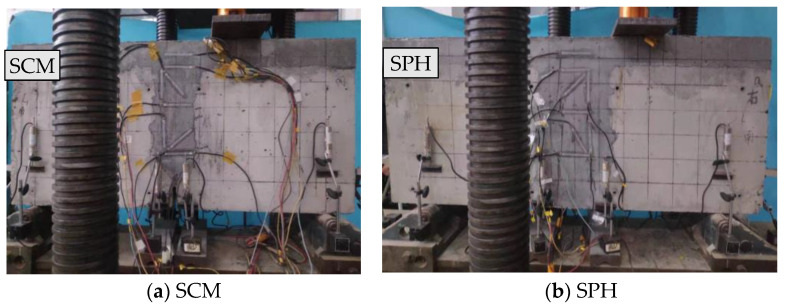
Damage of specimens [32].

The cracking loads of SCM and SPH were 130 and 180 kN, respectively, and the crack penetration loads were 205 and 280 kN, respectively. Their damage processes were the same as shown in Figure 3. The cracks mainly appeared at the joint surface of the loaded segment, and the specimens’ joint and failure processes were divided into two stages. The first stage showed no cracks in the specimen. At this stage, the stress of reinforcement in the joint and the relative deflections on both sides was zero, and the joint normally transmitted the load. The second stage was the cracking stage of the specimen. With the increase in load, the crack developed upward along the interface between the joint and the beam section, and the test stopped when the crack reached the lower edge of the pavement. At this stage, the stress of reinforcement and relative displacement grew with the increase in load.

## 3. Numerical Analyses of Specimens

### 3.1. FE Model

**Figure 4:**
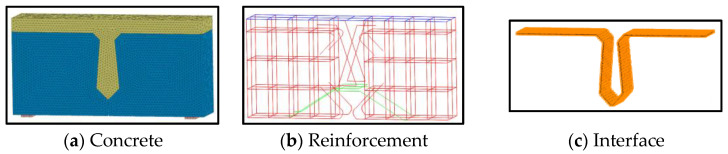
Diagram of FE model.

The finite element model, as shown in Figure 4, was established according to the design scheme of Section 2.1, and it included concrete, reinforcement, and interface. Considering the nonlinear finite element analysis, the meshing of the three-dimensional model was studied. The grid size would affect the accuracy of finite element analysis. The smaller the grid, the higher accuracy of the calculation results, but the speed of the analysis would also be greatly reduced. Under the premise of ensuring the accuracy of calculation, the grid size was determined to be 20 mm. Meanwhile, there were many irregular shapes in the joint. The tetrahedral grid shape can obtain high-quality solid elements at the corner of the joint, which is more conducive to the analysis and calculation.

Unlike other finite element software, the reinforcement unit in Midas FEA NX used embedded truss elements, which could be directly added to the structure. This kind of element only needed to set the basic material properties of the reinforcement, and the software would automatically handle the nodes of reinforcement and concrete so that the bond slip between the reinforcement and concrete did not need to be considered. In addition, when the interface elements were set, the nodes between the precast beam concrete element and the joint concrete element could be directly transferred from connection to disconnection.

### 3.2. Ontogenetic Relationship of Materials

#### 3.2.1. Concrete

**Figure 5:**
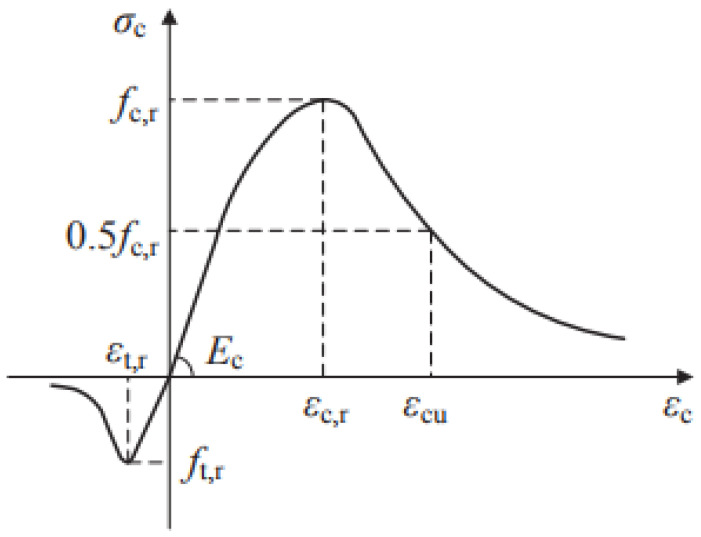
Stress-strain curve of concrete.

In MIDAS FEA NX, the main constitutive models for simulating the mechanical behavior of concrete were the concrete smeared crack mode and the concrete plastic damage model. The concrete plastic damage model (CDP) could reasonably simulate the cracking and damage of concrete materials. This study used this model to simulate concrete plastic material properties. The specific parameter settings are shown in Table 1. The uniaxial stress-strain curve of concrete is shown in Figure 5.

#### 3.2.2. Reinforcement

**Figure 6:**
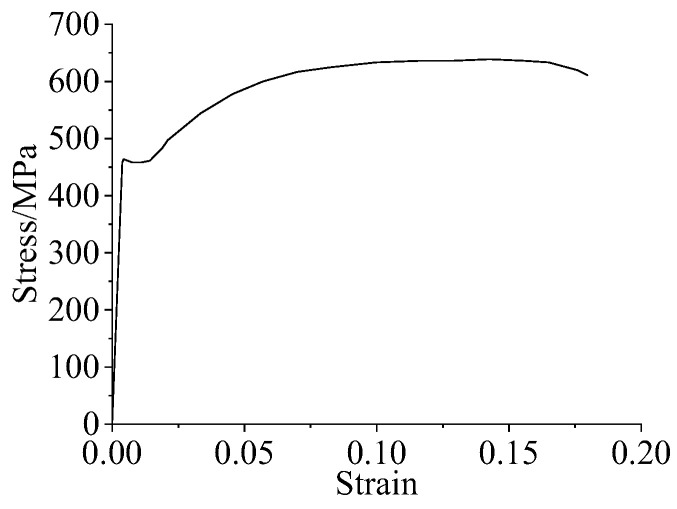
Tensile stress-strain curve of reinforcement.

The constitutive reinforcement model mainly included the isotropic linear elastic, Von Mises, and fully plastic models. Among them, the Von Mises model was widely used in the analysis of metallic materials, which mainly defined the same ductile material behavior as steel and could be used as an ideal elastic-plastic material for yielding damage when the stress reached a critical value and could also define the stress-strain curve independently. Therefore, the Von Mises model was used in this paper. According to reference [33], the uniaxial tensile stress-strain curve of reinforcement is shown in Figure 6, and the reinforcement parameters are shown in Table 2.

#### 3.2.3. Interface

**Figure 7:**
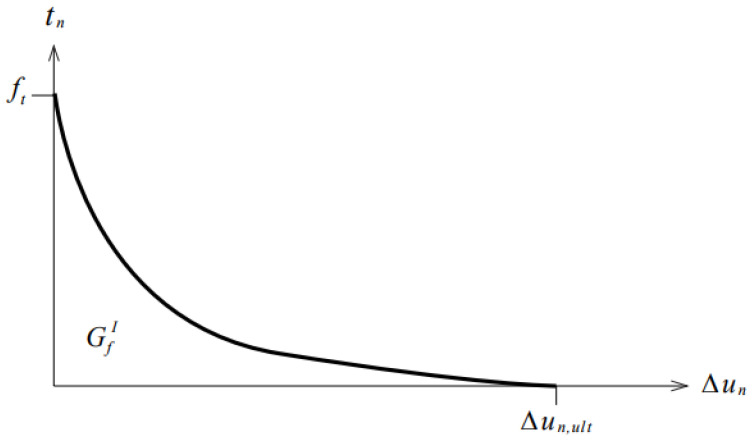
Nonlinear tensile softening constitutive relationship model.

According to the static test, the surface between the joint and the beam cracked, so the discrete cracked constitutive model was used to simulate the bond surface. The essence of this model was the combination of discrete elements and interface elements, and the relative displacement relationship between elements simulated the structural cracking. When the normal stress of the interface element reached the tensile strength, the relative displacement occurred between the main elements, equivalent to the cracking of the concrete bonding surface. In this paper, the nonlinear tensile softening model was selected as the functional relationship of the intrinsic model of the interface element (Figure 7), and the fracture energy in the model parameters was beneficial to the convergence of the calculation results.

The material parameters of the interface element were related to the mechanical properties of the structural material and needed to be adjusted according to the test results. The material parameters of the interface element included the normal stiffness modulus, tangential stiffness modulus, tensile strength, and fracture energy. The normal stiffness modulus was equal to the elastic modulus of concrete, and the tangential stiffness modulus was equal to 0.01 times the normal stiffness modulus. The tensile strength was 0.7 times that of the concrete axial tensile strength [34]. In the European regulation CEB-FIP 90, the calculation of fracture energy was as follows:

(1) $$G_{f}^{I}=a{\left(f_{c}/10\right)}^{0.7}$$

where *f_c_* is the sum of the concrete standard compressive strength, and 8 is called the average compressive strength; *a* = 0.025, 0.030, 0.058, corresponding to the maximum size of aggregate 8, 16, 32 mm, respectively.

### 3.3. Comparison of Experimental Results and Analysis Results

**Figure 8:**
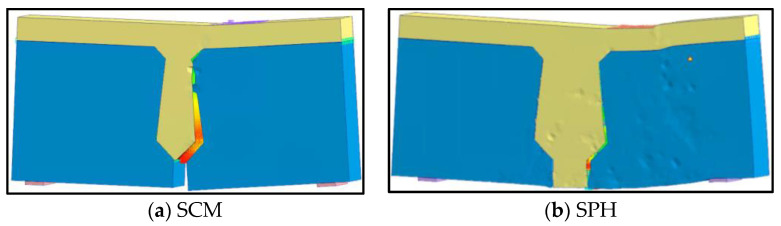
The failure mode of FEM.

Comparing the finite element analysis results of SCM and SPH with the experimental results (Table 3), it was found that the average value, standard deviation and coefficient of variation of crack penetration load were 0.91, 0.02 and 0.03, respectively, so the consistency between the experimental and analysis results of crack penetration load was high, with a maximum error of 12.20%. Moreover, the damage mode of the finite element model was shown in Figure 8, which was consistent with the location of specimen cracking in the statical test at the surface between the loading segment and joint. For the cracking load, SCM and SPH’s finite element analysis results were less than the experimental results. Because the transverse displacement of the interface element was the criterion in judging the crack load of the finite element model, but in the statical test, it could be judged by observing the cracks on the joint’s surface. Furthermore, the interface cracking inside the joint was earlier than the surface, and the cracking load of the finite element model was smaller than that obtained from the test.

**Figure 9:**
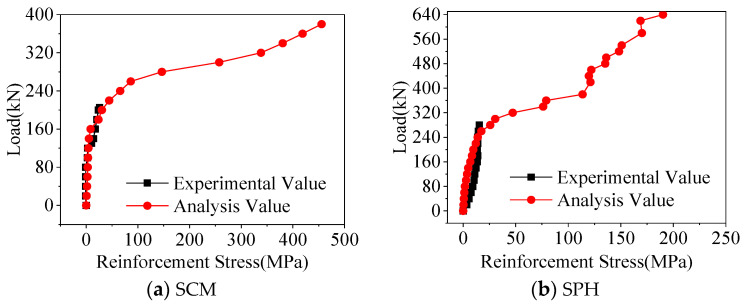
Comparison of reinforcement stresses.

As shown in Figure 9, the steel stresses of the finite element analysis and statical test were zero before the specimen cracking. After the specimen cracked, the steel stresses suddenly changed. Under the load of 200 kN, the maximum difference between the steel stresses on the loading beam segment of the finite element analysis and the statical test was 6.56 MPa, so the steel stresses were consistent.

**Figure 10:**
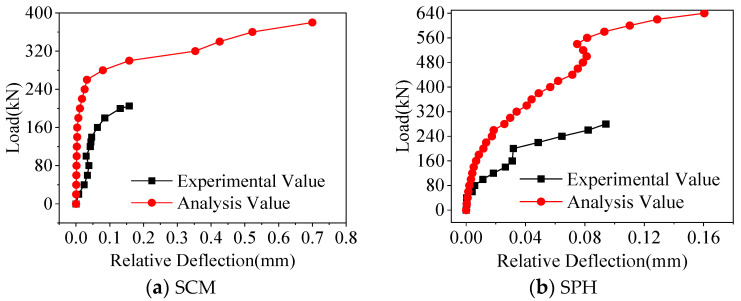
Comparison of relative deflection.

Figure 10 shows the comparison results of relative deflections. Before the crack penetration load, the relative deflection in the statical test was greater than that in the finite element analysis. This was because, in the finite element analysis, the parameters of the interface element were the same. However, in the statical test, the mechanical properties of the interface elements were inconsistent due to the limitations of the construction process, so there was an error in the relative deflection, and the maximum error was 0.07 mm.

In summary, Midas FEA NX could more accurately simulate the specimen with a joint by setting the interface element between the joint and beam. Through the comparative analysis of the results, this paper determined the interface parameters of the finite element models with different joints, and the specific values are shown in Table 3.

**Figure 11:**
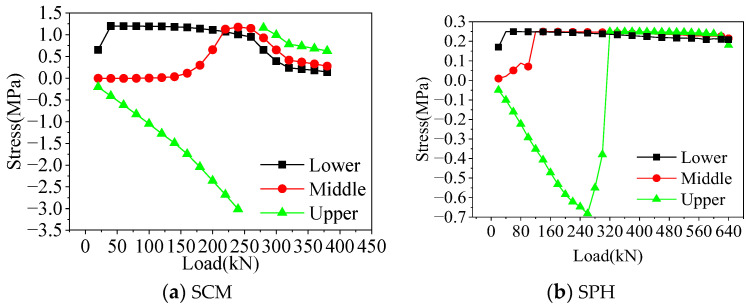
Interface stress.

Figure 11 shows the stress distribution of the interface element in the finite element analysis. Before the crack penetrating load, the interface element’s stress states at different positions differed. The lower and middle interface elements bore tensile stresses, while the upper interface elements bore compressive stresses. The stresses in the lower and middle interface elements continuously increased with the load increase. The interface element cracked when the stress was equal to the tensile strength in the material parameters. In addition, the compressive stress on the upper interface element increased at first, and when it reached the crack penetration load, the stress of the interface element suddenly changed to tensile stress and achieved the tensile strength value. The upper interface cracked at this time. From the perspective of the interface stress, it could be proved that the crack developed upward with the increasing load, which was consistent with the experimentally observed damage process. Therefore, the finite element analysis of SCM and SPH had a high degree of agreement with the experiment.

## 4. Numerical Analyses of Bridges

### 4.1. FE Model

**Figure 12:**
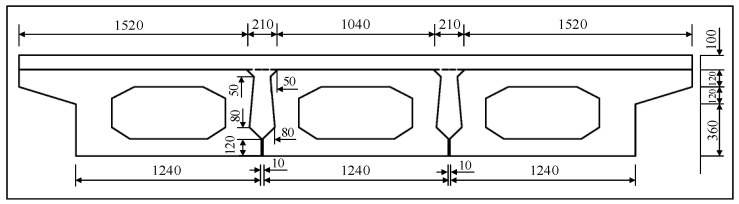
Detail of the hollow-core slab.

**Figure 13:**
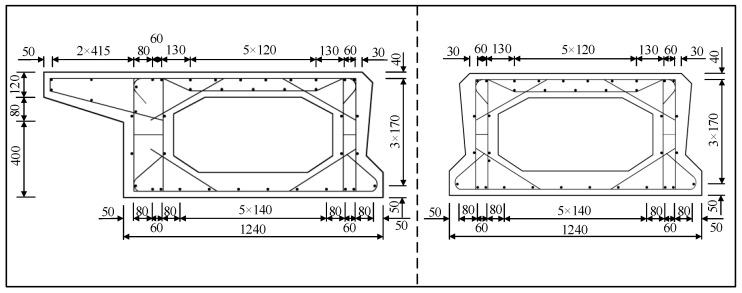
Hollow-core slab reinforcement details.

In this paper, a 10 m span hollow-core slab bridge was simulated by using Midas FEA NX. The hollow-core slab bridge adopted the bridge system of “three beams and two joints” [16] because the force of the joints gradually decreased with increasing distance from the loading point position. The hollow-core slab cross-section dimensions are shown in Figure 12, and reinforcement details are shown in Figure 13.

**Figure 14:**
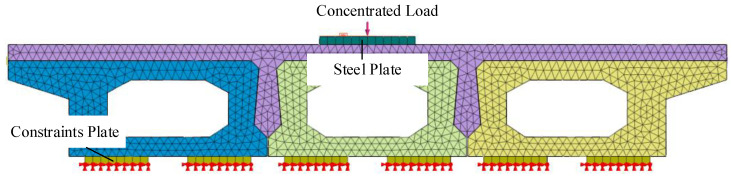
Diagram of hollow-core slab bridge grid.

Due to the extent limitation, the finite element model was presented in detail with the conventional reinforcement hollow-core slab bridge (BCM) model as an example. The finite element model of the bridge is shown in Figure 14. In order to facilitate the extraction of results, the 10 m hollow-core slab was divided into ten segments with 1 m as the cell length. The X-direction of the finite element model was the cross-sectional direction, the Y-direction was the longitudinal bridge direction, and the Z-direction was the vertical bridge direction. The finite element simulation assumes that there is no slip between the steel and concrete. Constraints were set at both ends of the longitudinal bridge direction, one side constrained the displacement of nodes in X, Y, and Z directions, and the other constrained the displacement of nodes in X and Z directions.

**Figure 15:**
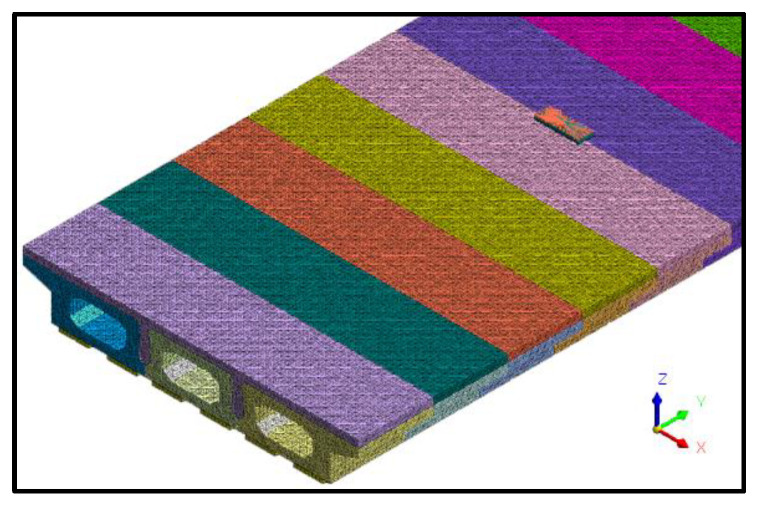
Diagram of hollow-core slab grid.

**Figure 16:**
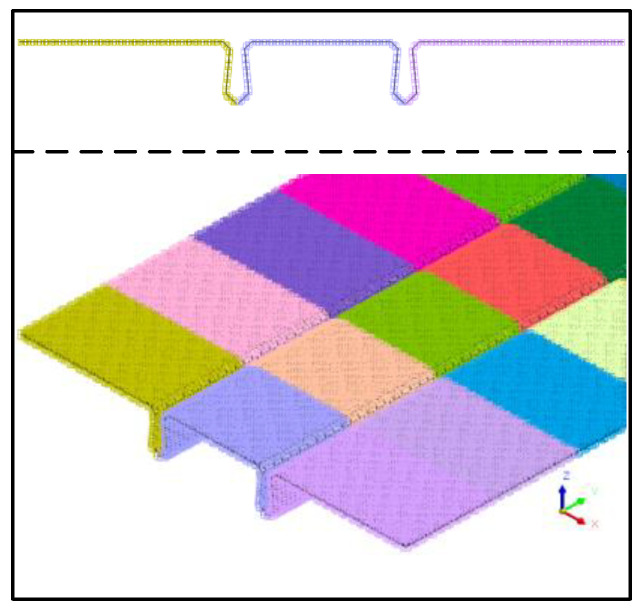
Interface unit.

To obtain more accurate nonlinear analysis results and to ensure computational efficiency, a tetrahedral mesh of size 50 mm was used to mesh the concrete entities, as shown in Figure 15, with a total of 602,270 elements for the hollow slab beam concrete entities and 240,470 elements for the pavement layer and joint concrete entities. Due to the contact relationship between the pavement layer, the joint, and the hollow-core slab, the contact between the old and new concrete was simulated using interface elements, and there was a total of 48,400 interface elements, as shown in Figure 16.

There was no slip damage of the reinforcement in the statical test, so implantable truss units were used to realize the common nodal coupling between the reinforcement elements and concrete elements with a size of 20 mm. According to the different diameters of reinforcements, hollow-core slab reinforcements and joint reinforcements were divided respectively, and there were 127,397 reinforcement elements in total.

### 4.2. Material Parameters

The elastic modulus of beam concrete was 32,599 N/mm^2^, and that of joint and pavement layer concrete was 35,992 N/mm^2^. Hot-rolled ribbed bars with a yield strength of 400 MPa were used for specimens, with an elastic modulus of 2 × 10^5^ MPa and Poisson’s ratio of 0.3. The interface element material parameters of the hollow-core slab bridge are shown in Table 4.

### 4.3. Result of Simulation

#### 4.3.1. Hollow-Core Slab Bridges with Conventional Reinforcement

**Figure 17:**
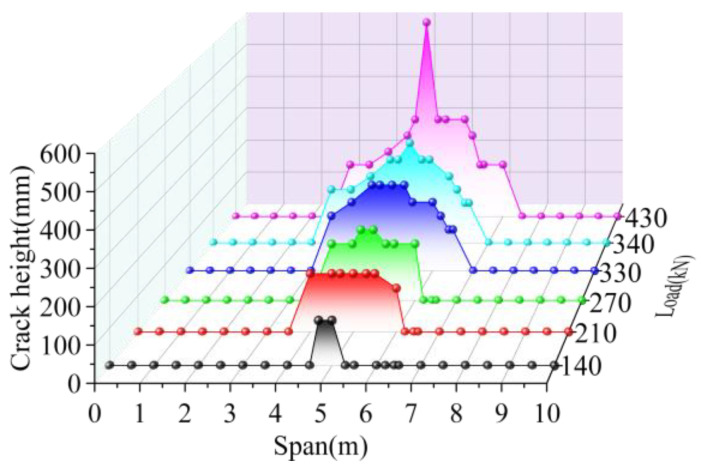
Interface crack height of BCM.

**Figure 18:**
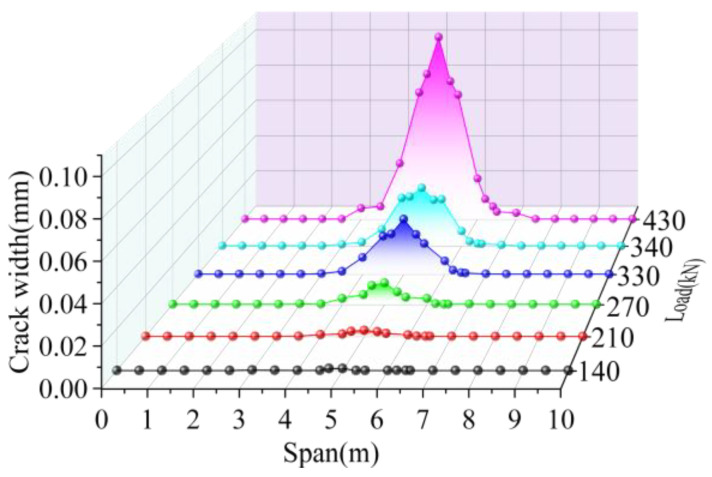
Interface crack width of BCM.

The cracking height of the joint interface elements is shown in Figure 17. The cracking width of the interface elements is shown in Figure 18. Under the load of 140 kN, the joint interface cracked at the load position; the crack height and width were 120 mm and 0.001 mm, respectively. The crack length of the interface along the longitudinal direction was 0.3 m. With the load increase, the cracks developed along the joint’s height direction and the bridge’s longitudinal direction. When the load reached 210 kN, the cracking height of the interface in the mid-span increased by 33.33%, its cracking width increased by 200%, and the cracking length in the longitudinal direction increased by 566.67%. When the load increased to 270 kN, the cracking height and width of the mid-span interface increased by 25.00% and 266.67%, respectively. When the load was increased to 330 kN, the crack height, crack width, and longitudinal crack length of the mid-span interface increased by 25.00%, 163.64%, and 50%, respectively; when the load increased to 340 kN, the crack of the interface along the longitudinal direction developed to 3/10 of the span, and the length increased by 16.67%. The crack height at the mid-span interface increased by 20.00%, and the crack width increased by 6.90. Under the ultimate load of 430 kN, the crack developed to the lower edge of the pavement layer, the maximum crack width in the mid-span was 0.102 mm, and the crack length in the longitudinal direction was 4 m. Comparing the data under the previous level of load, the crack height, crack length, and crack width increased 100.00%, 14.29%, and 229.03%, respectively.

In summary, the cracking started from the joint interface at the position where the load acts. The crack developed upward with the load increase. Finally, it reached the lower edge of the pavement layer. At the same time, the cracks at the joint interface developed symmetrically along the longitudinal direction and reached 0.3 and 0.7 times the span at last, and the crack length was 4 m. The crack width was the largest at the position where the load acts, and it decreased with the increase in distance from the load.

**Figure 19:**
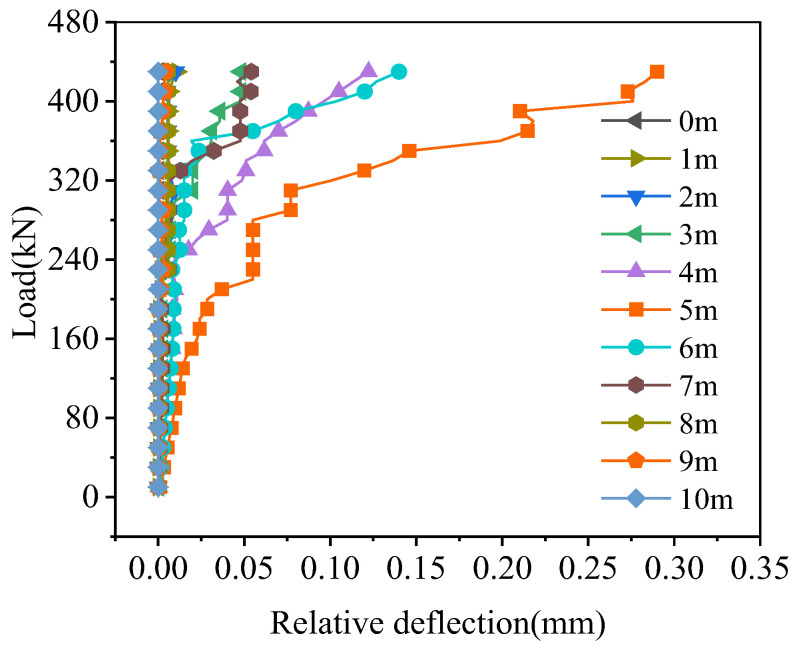
Relative deflection on joint of BCM.

The relative deflection of the hollow-core slab on both sides of the joint is shown in Figure 19. Except for the 0.3–0.7 times the span, the relative deflection at other locations was 0 because the damage of the joint interface at these locations was small; the interface crack width and crack height at these locations in Figure 17 and Figure 18 were equally small, and the joint interface was not cracked under the ultimate load. When the load reached 430 kN, compared to the relative deflections in the mid-span, the relative deflections at 0.3, 0.4, 0.6, and 0.7 times the span were reduced by 83.45%, 61.38%, 51.72%, and 81.38%, respectively. Therefore, the damage to the joint interface at the location where load acts in mid-span was the greatest, which resulted in the biggest relative deflection of the hollow-core slab, and the relative deflection decreased symmetrically with the increase of the distance from the load point.

**Figure 20:**
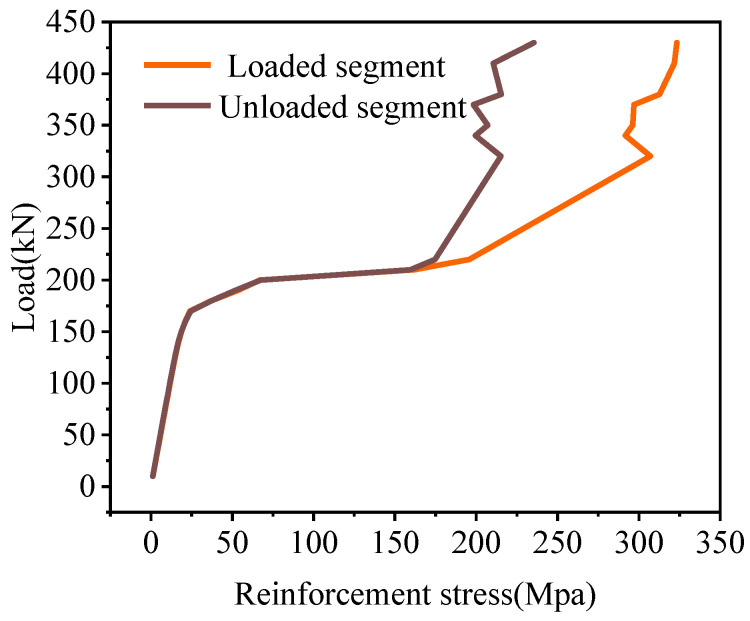
Joint reinforcement stress of BCM.

According to the location of the load, the hollow-core slab close to the load was called the loading segment, and the hollow-core slab far from the load was called the unloaded segment in this paper. The stresses of the tensile reinforcement in the joints are shown in Figure 20. The reinforcement stress increases with the load increase. After cracking the load, the stress of reinforcement on the loading segment was greater than that on the unloaded segment. Under the load of 340 kN, the stress of reinforcement on the loading segment was 1.96 times that on the unloaded segment. Overall, the tensile reinforcement stresses increased with the load increase, and the process showed a trend of uniform growth, rapid growth, and slow growth. Before the joint interface cracked, the stress of the tensile reinforcement was unchanged, and when the joint interface cracked, the load was mainly borne by the reinforcement. At that time, the stress of the tensile reinforcement grew rapidly. As the increase of load, the interface at the uncracked position could also transmit the load. At this time, the tensile reinforcement and the concrete in the joint bore the load together, so the reinforcement stress increased slowly.

#### 4.3.2. Hollow-Core Slab Bridges with Pinned Reinforcement and Grooves

**Figure 21:**
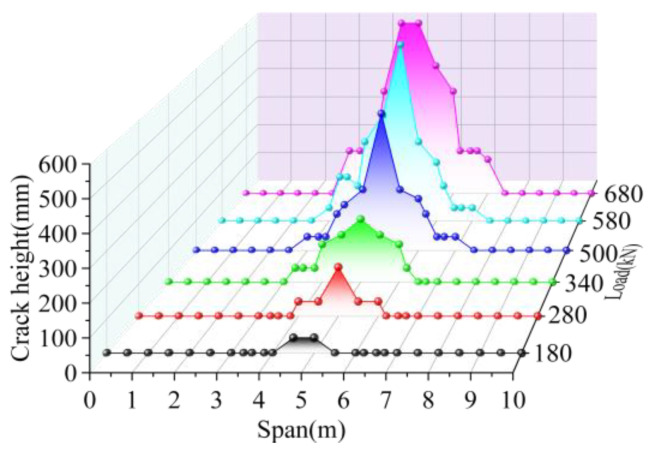
Interface crack height of BPH.

**Figure 22:**
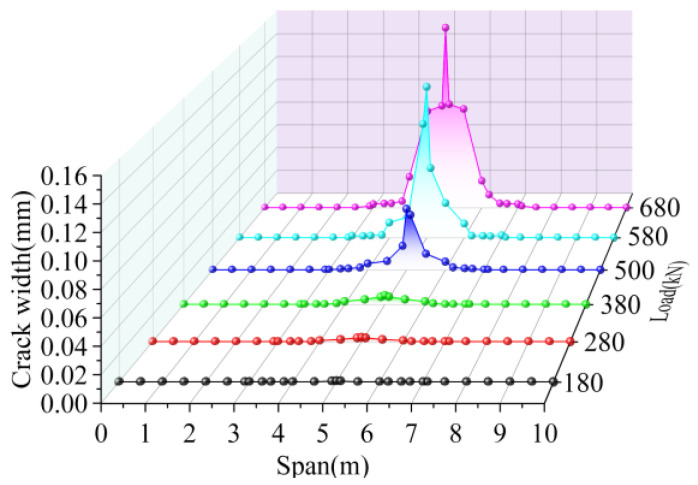
Interface crack width of BPH.

The cracking height of the joint interface elements is shown in Figure 21. The cracking width of the interface elements is shown in Figure 22. When the load was 180 kN, the hinge joint interface at the load position cracked with a crack height was 45 mm, the crack width was 0.001 mm, and the crack length was 0.2 m along the longitudinal direction. As the load increased, the crack height and width increased, and the interface at the remaining positions cracked, and when the load grew to 280 kN, the crack height of the joint interface at the mid-span load position increased by 233.33%, and the crack width increased by 200.00%. Along the longitudinal direction, cracks developed to 0.4 and 0.6 times the span, and the crack length increased by nine times. When the load increased to 380 kN, the crack height increased by 33.33%, the crack width increased by 133.33%, and the crack length increased by 16.67%. When the load increased to 500 kN, the cracking height of the joint at the mid-span increased by 125.00%, the crack width increased by six times, and the cracking length increased by 41.67%. When the load reached 580 kN, the crack height of the joint interface in the span was 600 mm, which increased by 33.33% compared with the crack height of the upper load, and the maximum crack width in the span increased by 157.17%. At this time, the crack at the joint interface developed to 0.3 and 0.7 times the span, and the longitudinal crack length reached 4 m. Under the ultimate load of 680 kN, the cracks developed in the lower edge of the pavement layer at the position 0.45 times the span, and the maximum crack width in the span increased by 23.02%. Therefore, under the load, the bridge was cracked at the load position. As the load increased, the cracks developed upward along the height and eventually reached the lower edge of the pavement layer. Cracks developed symmetrically toward the ends of the bridge along the longitudinal direction, eventually reaching 0.3 and 0.7 times the span, with a longitudinal crack length of 4 m. The crack width of the joint interface was greatest at the load position and gradually decreased symmetrically by increasing the distance from the load position.

**Figure 23:**
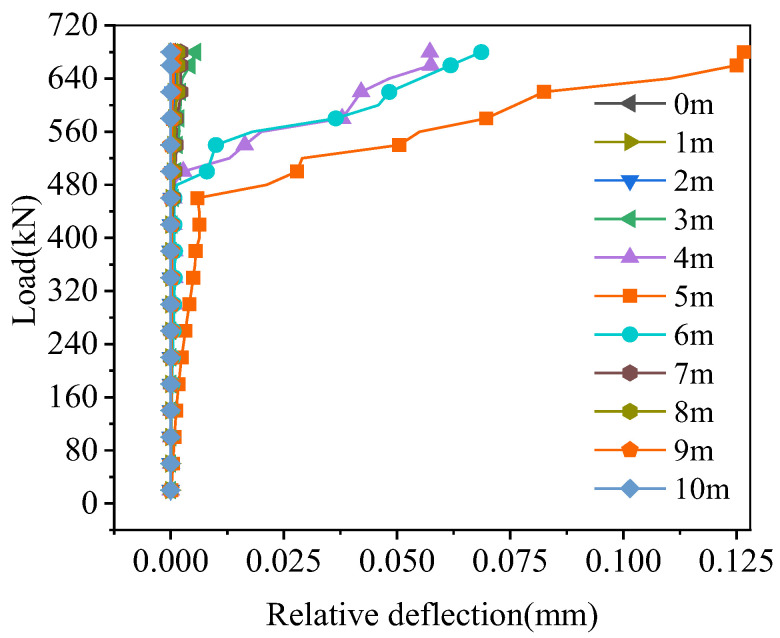
Relative deflection on joint of BPH.

The relative deflection of the hollow-core slab on both sides of the joint is shown in Figure 23. Except for the 0.2–0.8 times the span, the relative deflection at other locations was less than 0.01 mm. The relative deflection of the hollow-core slab was the largest at the load position and decreased with the increased distance from the load position. Under the load of 680 kN, the relative deflection of both sides of the joint at 0.5 times the span was 0.102 mm, and the relative deflection of the hollow-core slab at 0.3, 0.4, 0.6, and 0.7 times the span decreased by 98.04%, 31.37%, 43.14%, and 99.02%, respectively. Therefore, the damage to the joint interface at the load position was the greatest, and the relative deflection was the greatest.

**Figure 24:**
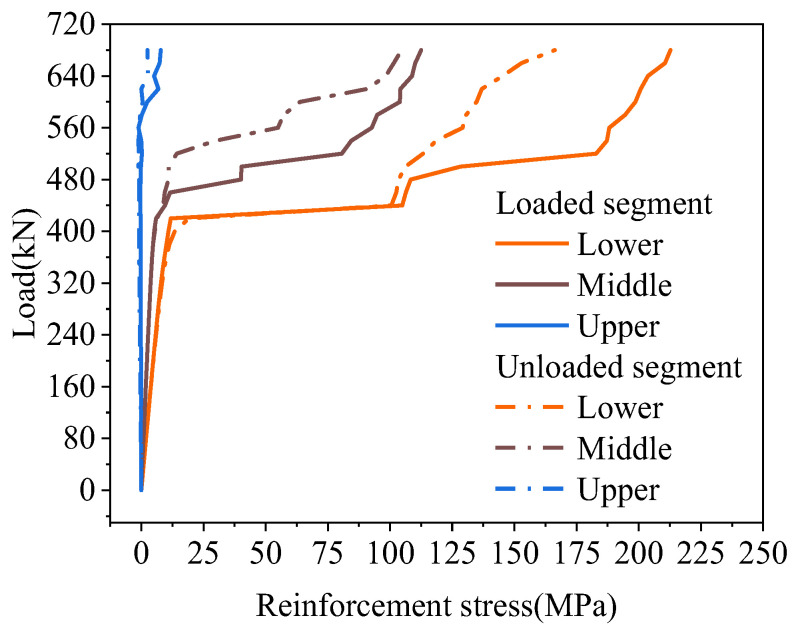
Reinforcement stress of BPH.

The stress trend of the tensile reinforcement in the joint is shown in Figure 24. Moreover, there were three layers of pinned reinforcement in the joint, and the stress of tensile reinforcement at the interface position was extracted. The stress of the lower reinforcement was the largest, and the stress decreased from bottom to top. Under the ultimate load of 680 kN, the stress of the lower reinforcement at the loading segment was 212.73 MPa. Compared with the lower reinforcement, the stress of the middle and upper reinforcement decreased by 47.11% and 96.32%, respectively. Furthermore, the stress of the lower reinforcement at the unloaded side was 166.23 MPa. Compared with it, the stress of the middle and upper reinforcement was reduced by 37.08% and 98.50%, respectively. The stress of the loaded segment was always greater than the unloaded segment because the load acted on the loaded segment, and the joint transferred the load from the loaded segment to the unloaded segment. The lower and middle reinforcement stress was tensile, which showed a uniform-rapid-slow change trend with the load increase. The upper reinforcement stress was compressive stress, and its stress was 0 MPa before the interface was cracked, and the reinforcement stress started to increase when the upper interface was cracked. Therefore, the reinforcement and the interface shared the role of load transfer, and after the interface cracking, the joint reinforcement at this location played the leading role.

### 4.4. Comparison of Different Bridges

#### 4.4.1. Bearing Capacity and Failure Mode

**Figure 25:**
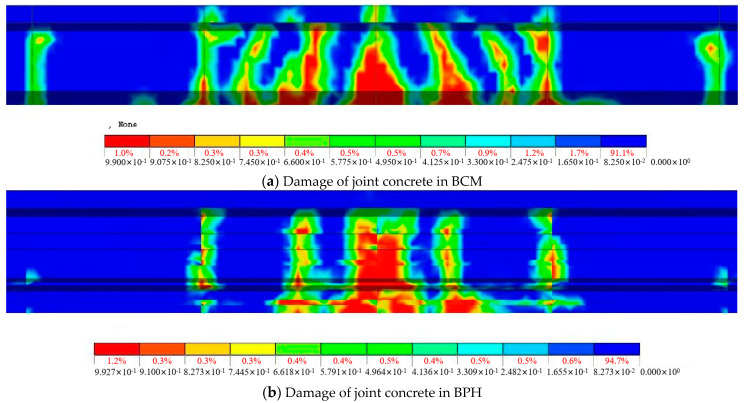
Diagram of joint concrete damage.

The bearing capacity of the conventional reinforcement hollow-core slab bridge (BCM) and pinned reinforcement hollow-core slab bridge (BPH) are shown in Table 5. The joint’s bearing capacity and damage mode were analyzed as an example. Figure 25 shows the damage to the joint concrete. 

For BCM, the bottom of the loading side joint interface at the load location was the first to crack when the load was 140 kN. After the cracks appeared, with the increase of load, the cracks developed along the height direction and longitudinal direction, and when the ultimate load of 680 kN was reached, the joint concrete of 0.3–0.7 times the span showed damages, and the damage range reached 4 m, which was consistent with the cracking length of the joint interface.

For BPH, when the load was 180 kN, the bottom of the joint interface on the loading segment cracked at the load location, and the cracking load was increased by 28.75% compared with BCM. With the load increase, the damage to joint concrete appeared. It could be seen from Figure 25 that under the ultimate load of 680 kN, the damage was mainly concentrated at the load position, and its damage range reached 2 m between 0.4–0.6 times the span, and compared with BCM, the ultimate load of BPH was increased by 58.14%.

#### 4.4.2. Reinforcement Stress

**Figure 26:**
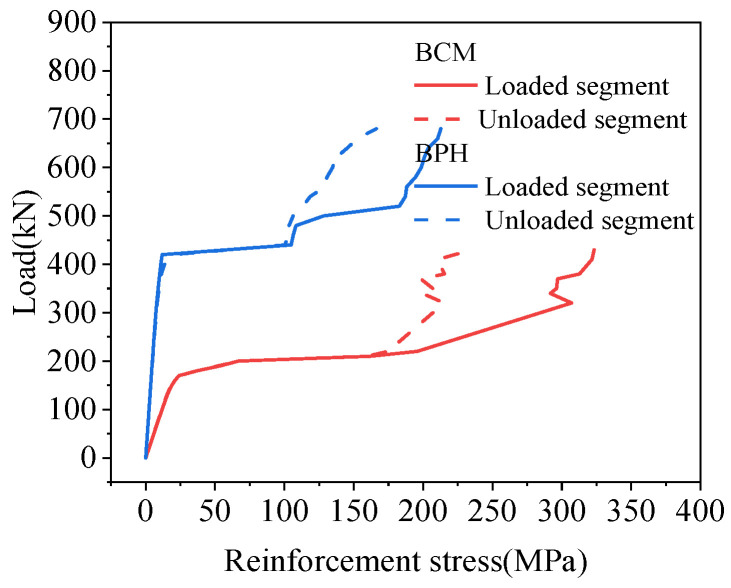
Comparison of reinforcement stress.

Under the load, the joint damage in the mid-span was the most serious, so the tensile reinforcement stresses in the lower part of the joint in BCM and BPH were shown in Figure 26. The tensile reinforcement stresses in the loaded segment of BCM and BPH were greater than the tensile reinforcement stresses in the unloaded segment. In contrast, the reinforcement stresses all showed a uniform-rapid-slow change trend. Under the same load, the tensile reinforcement stresses in the joint of different bridges were different. Under the load of 430 kN, the stresses in the loaded segment of BCM and BPH were 323.34 and 104.88 MPa, respectively, while the stresses in the unloaded segment were 235.44 and 100.51 MPa, respectively. Compared to the stresses in the BCM, the stresses in BPH’s loaded and unloaded segments were reduced by 67.56% and 4.17%. Therefore, changing the joint form could have a more significant effect on the reinforcement stresses, and the joint form with pinned reinforcement and grooves could enhance the reinforcement performance, reducing the stresses of tensile reinforcement at the bottom of the joint under the same load.

#### 4.4.3. Relative Displacement

**Figure 27:**
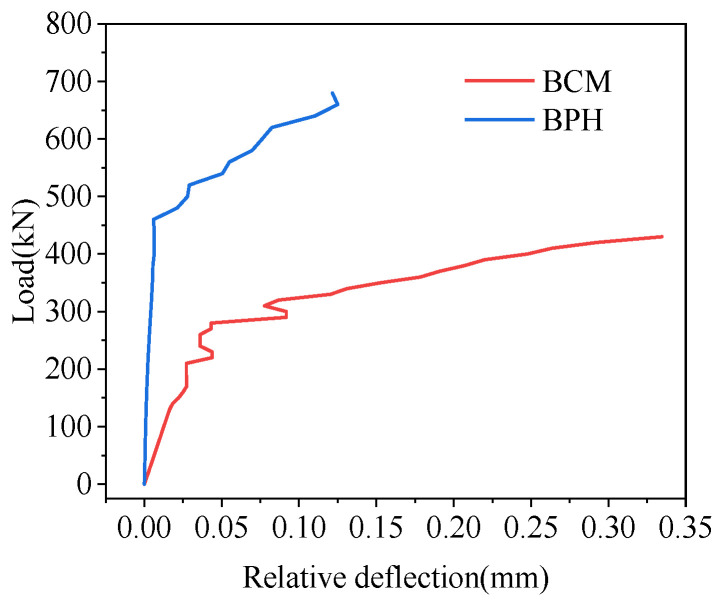
Comparison of relative deflection on joint.

The relative deflection of the hollow-core slab on both sides of the joint at the load position in mid-span is shown in Figure 27, which would increase with the load increase. Under the load of 420 kN, the relative deflection of the joint of BCM and BPH was 0.292 mm and 0.006 mm, respectively, and the relative deflection of BPH was 97.95% less than that of BCM. The results showed that using pinned reinforcement and grooves could enhance the bridge’s integrity and improve the joint’s load transfer performance. The grooves between the joint and the hollow-core slab enhanced the bonding effect, so this type of joint had good load transmission performance.

#### 4.4.4. Interface Crack Height and Width

**Figure 28:**
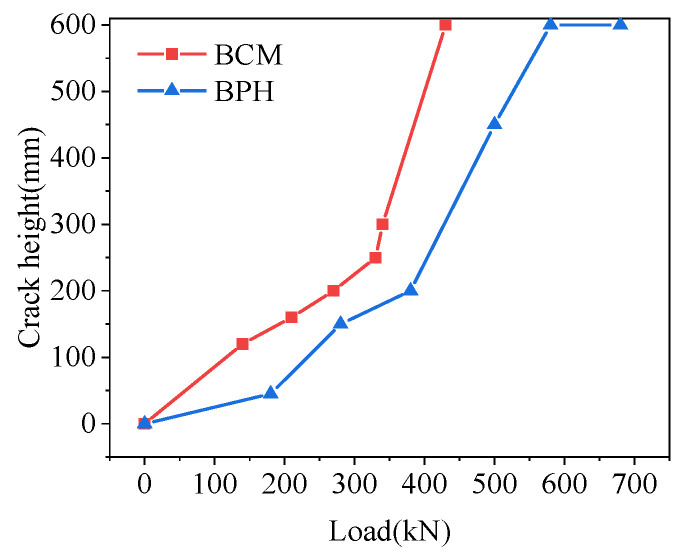
Comparison of interface crack height.

The crack height and width of the joint interface for hollow-core slab bridges with different joint forms under load are shown in Figure 28. Generally speaking, the crack height at the joint interface increases with the increase in load. Because different joint forms had different grids, the crack height growth trend was inconsistent. When the crack height reached 600 mm at the lower edge of the pavement layer, the corresponding loads of BCM and BPH were 430 kN and 580 kN, respectively, and the load of BPH was increased by 34.88% compared with BCM. Therefore, the hollow-core slab bridge with pinned reinforcements and grooves had better mechanical properties of joints and could slow down the development of cracks along the height direction.

**Figure 29:**
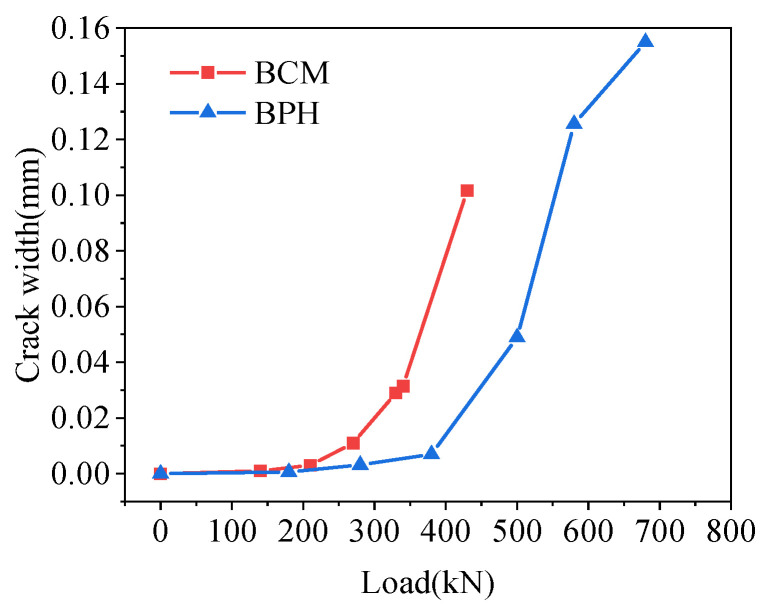
Comparison of interface crack width.

The crack width at the joint interface at the load position is shown in Figure 29. Overall, the crack width increased with the increase in load, and when the crack width at the joint interface reached 0.01 mm, the loads of BCM and BPH were 270 and 380 kN, respectively. Compared with BCM, the load of BPH increased by 40.74% at the same crack width, so the joint form of pinned reinforcement and groove slowed down the increase of crack width and improved the mechanical properties of joints.

## 5. Conclusions

In this study, a finite element model of a 10 m span assembled hollow slab bridge was established based on the hinge joint interface and concrete plastic damage unit characteristic parameters of the finite element analysis model of deep hinge joint section specimens to numerically analyze the whole bridge to explore the hinge joint damage evolution process and provide a basis for the improvement and strengthening of the hinge joint.

(1) The failure of the static load test and finite element analysis of the beam with deep joints is the cracking of the interface between the joint and the loaded segment. With the load increase, the cracking extends to the bottom of the pavement layer. According to the cracking situation, the force transmission mechanism of the joint can be divided into two stages: the uncracked stage and the cracked stage. In the uncracked stage, the joint surface mainly transfers load, and the stress of the tensile reinforcement at this position is minor. In the cracked stage, the bonding force of the joint surface at the cracking location disappears, and the bonding force between the reinforcement and concrete and the reaction force of the concrete column jointly transfer the load.
(2) Compared with the results of finite element analysis and statical load test, the stress and relative deflection of reinforcement are the same, and the maximum error of load penetrating crack is 12.2%. The failure of the finite element model is mainly the cracking of the interface between the joint and the hollow-core slab. Before the crack penetration load, the joint surface presents the stress distribution of tension in the lower part and compression in the upper part, and the height of the tension zone increases gradually with the increase in load.
(3) A finite element model of a 10 m span assembled hollow-core slab bridge is established using material parameters of the beam segment with deep joint to study the bridge’s force transfer performance and damage evolution. The results show that the failure of hollow-core slab bridge starts from the joint interface at the load location, and with the load increase, the damage to the joint appears, and the damage range of convention reinforcement hollow-core slab bridge under ultimate load is 3–7 m. The cracking develops along the height and longitudinal directions with the load increase, and the cracking height can reach 600 mm. The cracking length of the convention reinforcement joint and pinned reinforcement joint hollow-core slab bridge can get 4 and 2 m. The damage to the joint interface is most profound at the load location, where the interface cracking height, crack width, and relative deflection are the largest, and they decrease with the increase of distance from the load location. The stress of the tensile reinforcement in the hinge joint shows a trend of rapid growth, slow growth, and growth with the load increase.
(4) Analysis of the bearing capacity, joint reinforcement stress, relative deflection, cracking height, and crack width of hollow-core slab bridges with different joints shows that the pinned reinforcement bridges have better mechanical behavior, and the ultimate load is increased by 62.79% compared with the conventional reinforcement bridges. Compared with the conventional reinforcement hollow slab bridge, when the load reached 400 kN, the stress and relative deflection of the pin-connected steel hollow slab bridge hinge joint tensioned reinforcement decreased by 96.77% and 97.58%, respectively; when the hollow slab bridge hinge joint interface cracking height developed to 600 mm, the load of the pin-connected steel hollow slab bridge increased by 34.88%; when the crack width reaches 0.01 mm, its load increases by 40.74%. Therefore, the hinge joint form of pin jointed reinforcement with groove can improve the interface bond, delay the development of hinge joint interface cracks, enhance the transverse force transfer performance of the hinge joint and the integrity of the hollow slab bridge, and then improve the force performance of the hollow slab bridge.
(5) In fact, we do not find suitable results from other scholars to compare. In our subsequent study, we will continue to investigate the mechanical properties of other forms of hollow slab bridges and analyze them in comparison with the two bridges in this paper. Meanwhile, it is challenging to conduct load tests due to the large size of full-size bridges, so this study needs tests for full-size bridges. We will complete this work in a future study.

## Figures and Tables

**Table 1:** Concrete parameter table.

Parameter	Elastic Modulus (N/mm^2^)	Poisson’s Ratio	Expansion Angle	Eccentricity	fb/fc	Kc	Sticky Parameters
Value	29,791	0.20	30	0.10	1.10	0.67	0.0001

**Table 2:** Reinforcement parameter table.

Parameter	Elastic Modulus (N/mm^2^)	Poisson’s Ratio	Bulk Density (N/mm^3^)	Damping Ratio
Value	200,000	0.30	7.698 × 10^−5^	0.01

**Table 3:** The results of bearing capacity.

Specimens	Cracking Load (kN)			Crack Penetration Load (kN)			Ultimate Load (kN)
	Experimental Results	Analysis Results	Error	Experimental Results	Analysis Results	Error	Analysis Results
SCM	130	60	2.17	205	230	0.89	380
SPH	180	120	1.50	280	300	0.93	640
Average value	-	-	1.8	-	-	0.91	-
Standard deviation	-	-	0.3	-	-	0.02	-
Coefficient of variation	-	-	0.3	-	-	0.03	-

**Table 4:** Interface material parameters.

Bridge	Joint Form	Normal Stiffness Modulus (N/mm^3^)	Tangential Stiffness Modulus (N/mm^3^)	Tensile Strength (N/mm^2^)	Fracture Energy (N/mm)
BCM	Conventional reinforcement	15,000	150	1.20	0.72
BPH	Pinned reinforcement and grooves	15,000	150	0.25	0.72

**Table 5:** Bearing capacity of hollow-core slab bridges in different joint forms.

Bridge	Joint Form	Cracking Load (kN)	Ultimate Load (kN)
BCM	Conventional reinforcement	140	430
BPH	Pinned reinforcement and grooves	180	680

## Data Availability

Data requirements can be directed to corresponding author.
